# Nutritional interventions for the treatment of frailty in older adults: a systematic review protocol

**DOI:** 10.1097/MD.0000000000013773

**Published:** 2018-12-28

**Authors:** Mariana Bordinhon de Moraes, Carolina Fumico Massuda Araujo, Christina Avgerinou, Edison Iglesias de Oliveira Vidal

**Affiliations:** a Department of Public Health; b Internal Medicine Department, São Paulo State University (UNESP), Botucatu Medical School, SP, Brazil; c Department of Primary Care and Population Health, University College London, London, UK.

**Keywords:** aged, diet, dietary supplements, feeding, frailty, nutrition, systematic review

## Abstract

**Background:**

Frailty has been defined as a clinical syndrome of multicausal origin characterized by a reduction of physiologic reserves that increase the vulnerability of an individual to adverse outcomes such as the development of functional dependence and death. Considered one of the most important geriatric syndromes, frailty's prevention and management represent important goals for gerontology and geriatrics. Although nutrition plays an important role within the multifactorial susceptibility for this syndrome, up to the present no systematic review specifically addressed the effectiveness of nutritional interventions for the treatment of frailty. Therefore, we propose the present systematic review with the aim to assess the effectiveness of nutritional interventions for the treatment of frailty in older adults living in the community or in long-term care facilities.

**Methods:**

We will search Medline (via Pubmed), Embase, Cinahl, Central, Lilacs, Web of Science, and sources of gray literature. We will accept trials whereby the unit of randomization consisted of individuals or clusters of individuals. Our primary outcome is all-cause mortality. Secondary outcomes are quality of life, functional status, cognitive function, frailty status, body composition, and physical activity. Risk of bias will be assessed using the Cochrane Collaboration tool. We will analyze the overall strength of the evidence for each outcome using the Grading of Recommendations Assessment, Development and Evaluation (GRADE) tool. Two independent researchers will conduct all evaluations and any disagreements will be resolved through the participation of a 3rd author. If possible, we will perform random-effects meta-analyses and subgroup analyses concerning specific details of nutritional interventions (e.g., components and duration), research scenario, risk of bias, and criteria used to diagnose frailty.

**Conclusion:**

In this systematic review protocol we outline the details of the aims and methods of a systematic review on the effectiveness of nutritional interventions for the management of frailty in older adults living in the community or in long-term care facilities”. We believe this wording to be more objective and balanced than the previous one. We understand that it is not ideal to propose changes to the text after manuscripts have been accepted. However, we feel that the new wording of the conclusion section of the abstract is more consistent with the overall content of the main text of the review than its previous version. Hence, we hope you may accept our request.

## Abstract 2

Supplemental Digital Content is available in the text

## 1 Introduction

Frailty has been defined as a clinical syndrome of multicausal origin characterized by a reduction of physiologic reserves that increase the vulnerability of an individual to adverse outcomes such as the development of functional dependence and death.^[1]^

Considered one of the most important geriatric syndromes, frailty's prevention and management represent important goals for gerontology and geriatrics.^[2]^ The concept of frailty has greatly contributed to the development of this field by highlighting a multiplicity of subclinical factors (i.e., going beyond the presence of functional dependence and comorbidities) and contributing to the reduction of the capacity of older adults to maintain their homeostasis when exposed to stressor events.^[3]^ In fact, studies using different operational definitions of frailty have shown that it represents an important risk factor for a variety of negative outcomes. For example, frail older adults were found to be at an increased risk of falling by 84%, when compared to those who are nonfrail.^[4]^ The frailty syndrome has also been associated with 70% greater chance of fractures,^[5]^ 30% increase in the risk of developing dementia,^[6]^ and 90% increase in the risk of hospitalization.^[7]^ An inverse association between frailty and quality of life of older adults living in the community has also been observed.^[8]^

These data are especially relevant when one considers the results of studies reporting the prevalence of this syndrome among older adults and the perspectives of population aging worldwide.^[9]^ A systematic review on the prevalence of frailty among community-dwelling elderly identified that prevalence ranged from 4% to 59%, with a weighted average of 11%.^[10]^ A significant increase in the prevalence of this syndrome is also noted among individuals of a more advanced age, reaching an average of about 27% among adults older than 85 years of age.^[11]^ Amid institutionalized older adults, the prevalence of frailty ranged from 19% to 76%, with a weighted average of 52%.^[11]^

An important meeting of experts, leading to the 1st successful international consensus on the definition of frailty, considered that there was some evidence suggesting possible benefits of 4 types of interventions for managing this condition: physical exercise, caloric and protein support, vitamin D supplementation, and reduction of polypharmacy.^[1]^

Loss of muscle mass is one of the consequences of weight loss in older adults, along with reduction of strength, mobility, and immune dysfunction, which represent typical characteristics of frailty. In addition, malnutrition in older adults increases the risk of hospitalization, functional dependence, and death in this population.^[12]^ The association between nutritional factors and the occurrence of frailty was also observed in the systematic review of Lorenzo-López et al that analyzed data from 19 observational studies.^[13]^ The nutritional factors examined by this review were micronutrients, macronutrients, diet quality, antioxidants, and score in the Mini Nutritional Assessment.^[13]^

Due to the global phenomenon of population ageing,^[2]^ the increased prevalence of frailty at more advanced ages and the negative consequences of this syndrome, studies about efficacy and effectiveness of interventions to manage this syndrome have great importance, particularly aiming at the prevention of such adverse events. In view of the relevance of the topic and the arguments presented above, we propose the present systematic review with the aim to assess the effectiveness of nutritional interventions for the treatment of frailty in older adults living in the community or in long-term care facilities.

## 2 Methods

### 2.1 Study registration

This systematic review protocol has been registered on PROSPERO under the number of CRD42018111510, and was performed in accordance with the Preferred Reporting Items for Systematic Reviews and Meta-analysis Protocol.^[14]^ This is a literature-based study, so ethical approval is unnecessary.

### 2.2 Selection criteria

#### 2.2.1 Types of studies

We will include only parallel-group randomized clinical trials published since 2001 in English, Portuguese, or Spanish. We will accept trials whereby the unit of randomization consisted of individuals or clusters of individuals.

#### 2.2.2 Types of participants

We will include studies that recruited older adults (aged 60 years or older) with a diagnosis of frailty or prefrailty and living in the community or in long-term care facilities. We will accept any criteria used by original studies to diagnose that syndrome. Studies that have been performed during hospitalization episodes will not be included.

#### 2.2.3 Types of interventions

We will include studies that have implemented at least one of the following nutritional interventions: nutritional education/dietary prescription, the use of hypercaloric or hyperproteic dietary oral supplements and the delivery of specific diets. Additionally, we will also include studies that adopted any of the above interventions concomitantly with another single or multifactorial intervention provided that the comparator was the same set of interventions without the nutritional intervention component. We will accept as comparators standard treatment, placebo, other nutritional interventions, and multifactorial interventions without a nutritional component.

#### 2.2.4 Types of outcomes

We will include studies if they report at least one of the following outcome measures.

##### 2.2.4.1 Primary outcomes

1. Mortality.

##### 2.2.4.2 Secondary outcomes

1. Quality of life, measured by any instrument
2. Functional capacity, measured by any instrument.
3. Cognitive function, measured by any instrument.
4. State of frailty, measured by any instrument.
5. Body composition, measured by any instrument.
6. Physical activity, measured by any instrument.

### 2.3 Search methods for study identification

Two independent researchers will examine the lists of references identified through electronic search. We will also hand-search reference lists of relevant publications including review articles on frailty and of original studies considered eligible for the review. Additionally, we will contact experts in the field of nutrition and frailty to ask for references to published and unpublished data. We also intend to contact researchers to request relevant unpublished data whenever possible.

#### 2.3.1 Electronic searches

We will search the following databases for relevant studies, using the search terms detailed in Appendix 1: Medline (via Pubmed), Embase, Cinahl, Central, Lilacs e Web of Science.

#### 2.3.2 Other resources

We will search the following databases for gray literature: System for information on Gray Literature in Europe (Open Gray), Virginia Henderson Global Nursing e-Repository, National Library of Medicine Bookshelf, ClinicalTrials.gov, and World Health International Clinical Trials Registry Platform (ICTRP).

### 2.4 Data collection and analysis

#### 2.4.1 Selection of studies

For all studies identified, 2 authors will independently screen and review the titles and abstracts. Full versions of potentially relevant studies will be obtained. Where applicable, we will contact the authors of selected studies to ask for additional data. Disputes regarding the inclusion of a study will be resolved through discussion with a 3rd reviewer.

### 2.5 Data extraction and management

Two reviewers will extract data independently using a standardized prepiloted form including the following data: complete reference; time period when the study was conducted; geographical location; presence of divergences between the study protocol and published results; study design; types of interventions and comparators; duration of the intervention and of follow-up; inclusion/exclusion criteria; sample size; characteristics of the population; balance between groups at the baseline; funding source; method of randomization; presence of simultaneous interventions; diagnostic criteria of frailty; nutritional interventions; details of the intervention, including type, dose, frequency, and duration; control treatment; outcome measures; blinding (patients, field professionals and outcome assessors); duration of follow-up; loss of follow-up; results; intention-to-treat analysis; conclusions reported by the study authors; and research limitations. In addition, there will be a field for the registration of other information deemed relevant by the reviewers.

Disagreements about extracted data will be resolved by consensus, and an independent reviewer will be consulted if disagreement persists.

#### 2.5.1 Assessment of bias risk

To assess the risk of bias in the included studies, 2 review authors will independently use The Cochrane Collaboration's Risk of Bias tool for randomized clinical trials.^[15]^ Accordingly, the following domains will be assessed: random sequence generation, allocation concealment, blinding, incomplete outcome data, selective reporting, and other bias. Each of these criteria will be assigned one of the following categories: low risk of bias; high risk of bias; or unclear risk of bias, where unclear relates to the lack of precise information or uncertainty over the potential for bias.

Where applicable, the investigators of selected trials will be contacted to provide additional relevant information. Disagreements between the authors regarding the assessment of risk of bias will be resolved by consensus, and a 3rd reviewer will be consulted when needed.

#### 2.5.2 Rating quality of evidence

We will analyze the overall strength of the evidence for each outcome using the Grading of Recommendations Assessment, Development and Evaluation (GRADE) tool. This system represents a method that evaluates the quality of evidence in systematic reviews explicitly, comprehensively, transparently, and pragmatically.^[16]^ The GRADE system evaluates the following dimensions regarding the quality of evidence: study limitations/risk of bias, inconsistency, indirect effects, inaccuracy, publication bias, and factors that may increase the quality of evidence. According to GRADE, the quality of the evidence regarding each outcome analyzed is classified into 1/4 levels: high, moderate, low, and very low.^[16]^

We will use the GRADE profiler software (GRADEPRO) to create “summary of findings” tables with outcome specific information concerning the overall quality of evidence and the magnitude of effect of the interventions examined by the examined body of evidence.

#### 2.5.3 Measures of treatment effects

Dichotomous data: the results will be presented as the risk ratios with 95% confidence intervals. Continuous data: the results will be presented as the mean difference, if outcomes are measured using similar scales between trials. We will use the standardized mean difference to combine trials that measure the same outcome using different scales or instruments.

#### 2.5.4 Unit of analysis issues

The appropriate unit of analysis will be the individual patient, rather than hospitals or health centers. In studies with multiple intervention groups, we will include only the comparisons between groups that meet our eligibility criteria. If more than 1 pair of intervention comparisons are eligible for a given meta-analysis and those pairs of comparisons have at least 1 intervention group in common, we will proceed using one of the methods recommended by the Cochrane Collaboration in the following order of preference according to the feasibility of each approach: we will attempt to merge the intervention groups to yield a single pairwise comparison; we will attempt to account for the correlation between correlated comparisons by calculating a weighted average of the different pairwise comparisons; and we will perform a network meta-analysis.

#### 2.5.5 Missing data

Where applicable, we will contact the chief investigators of clinical trials with missing data or unclear information (e.g., unclear risk of bias). Whenever possible we will include in meta-analyses data from intention-to-treat analyses. We will not perform imputation procedures for missing data.

#### 2.5.6 Assessment of reporting biases

If there are sufficient numbers of trials (at least 10), we will construct a funnel plot and we will apply the Egger tests and the Trim and Fill method in the evaluation of publication bias.

#### 2.5.7 Data synthesis

We will organize the synthesis of data according to the types of nutritional interventions studied, the types of comparators, and populations studied (i.e., older adults living in the community or in long-term care facilities).

If the included studies are sufficiently similar in terms of population, inclusion criteria, interventions, and results, we will perform quantitative synthesis using the random effects models.

#### 2.5.8 Assessment of heterogeneity

If the available data allow the performance of meta-analyses, we will assess statistical heterogeneity by means of *I*^2^ statistics, which will be interpreted according to the current Cochrane Collaboration guidance as follows: 0% to 40% might not be important; 30% to 60% may represent moderate heterogeneity; 50% to 90% may represent substantial heterogeneity; 75% to 100% considerable heterogeneity.^[15]^ If we find substantial heterogeneity, we will attempt to perform subgroup analyses as described in the following sections.

#### 2.5.9 Subgroup analyses

If sufficient data are available, we will perform the following subgroup analyses: concerning specific details of nutritional interventions (e.g., components and duration), research scenario (i.e., community or long-term care facilities), risk of bias, and criteria used to diagnose frailty.

#### 2.5.10 Sensitivity analysis

We have not planned any sensitivity analyses.

## 3 Discussion

Nutrition plays an important role within the multifactorial susceptibility of this syndrome; however, up to the present no systematic review addressed the effectiveness of nutritional interventions for the treatment of frailty. The systematic specifically reviews identified in the literature on this topic emphasize interventions related to physical activity without any particular focus to nutritional interventions, which were generally analyzed briefly and in a secondary manner.^[9,13,17–22]^

## Author contributions

**Conceptualization:** Mariana Bordinhon de Moraes, Edison Iglesias de Oliveira Vidal.

**Data curation:** Mariana Bordinhon de Moraes, Edison Iglesias de Oliveira Vidal.

**Formal analysis:** Mariana Bordinhon de Moraes, Christina Avgerinou, Edison Iglesias de Oliveira Vidal.

**Investigation:** Mariana Bordinhon de Moraes, Carolina Fumico Massuda Araujo, Edison Iglesias de Oliveira Vidal.

**Methodology:** Mariana Bordinhon de Moraes, Carolina Fumico Massuda Araujo, Edison Iglesias de Oliveira Vidal.

**Project administration:** Mariana Bordinhon de Moraes, Christina Avgerinou, Edison Iglesias de Oliveira Vidal.

**Resources:** Mariana Bordinhon de Moraes, Christina Avgerinou, Edison Iglesias de Oliveira Vidal.

**Supervision:** Edison Iglesias de Oliveira Vidal.

**Validation:** Mariana Bordinhon de Moraes, Carolina Fumico Massuda Araujo.

**Visualization:** Mariana Bordinhon de Moraes, Carolina Fumico Massuda Araujo.

**Writing – original draft:** Mariana Bordinhon de Moraes, Carolina Fumico Massuda Araujo.

**Writing – review & editing:** Mariana Bordinhon de Moraes, Christina Avgerinou, Edison Iglesias de Oliveira Vidal.

## Supplementary Material

Supplemental Digital Content
