# Nutritional interventions for the treatment of frailty in older adults: a systematic review protocol: Erratum

**DOI:** 10.1097/MD.0000000000014358

**Published:** 2019-01-25

In the article, “Nutritional interventions for the treatment of frailty in the older adults a systematic review protocol”,^[1]^ which appeared in Volume 97 Issue 52 of *Medicine*, there was a publisher error in which additional comments were added to the paper's abstract conclusion section.

The abstract's conclusion should read: “In this systematic review protocol we outline the details of the aims and methods of a systematic review on the effectiveness of nutritional interventions for the management of frailty in older adults living in the community or in long-term care facilities.”

In addition, the word “specifically”’ was misplaced in the discussion section. The discussion section should read: “Nutrition plays an important role within the multifactorial susceptibility of this syndrome; however, up to the present no systematic review specifically addressed the effectiveness of nutritional interventions for the treatment of frailty. The systematic reviews identified in the literature on this topic emphasize interventions related to physical activity without any particular focus to nutritional interventions, which were generally analyzed briefly and in a secondary manner.^[9,13,17–22]^”

Finally, the full affiliation of Mariana Bordinhon de Moraes and Carolina Fumico Massuda Araujo should read: Department of Public Health, São Paulo State University (UNESP), Botucatu Medical School, SP, Brazil.
