# Supplementary material for: Nutritional interventions for the treatment of frailty in older adults: a systematic review protocol
Source: Medicine (Baltimore). 2018 Dec 28;97(52):e13773. doi: 10.1097/MD.0000000000013773 (PMC6314758; doi:10.1097/MD.0000000000013773)
Supplement: Supplemental Digital Content [file medi-97-e13773-s001.docx]

**Appendix 1**

1. **Medline**

| Search ID # | Search formula |
| --- | --- |
| 1 | (Frailty [Mesh] or Frailties [all fields] or Frailness [all fields] or Frailty Syndrome [all fields]) |
| 2 | (Aged [Mesh] or Elderly [all fields) or Senior [all fields]) |
| 3 | (Geriatrics [Mesh] or Geriatric [all fields]) |
| 4 | (Aged, 80 and over [Mesh] or Oldest Old [all fields] or Nonagenarians [all fields] or Nonagenarian [all fields] or Octogenarians [all fields] or Octogenarian [all fields] or Centenarians [all fields] or Centenarian [all fields]) |
| 5 | (Frail Elderly [Mesh] or Frail Elders [all fields] or Frail Elder [all fields] or Functionally-Impaired Elderly [all fields] or Functionally Impaired Elderly [all fields] or Frail Older Adults [all fields] or Frail Older Adult [all fields]) |
| 6 | 2 or 3 or 4 or 5 |
| 7 | (Dietary Supplements [Mesh] or Dietary Supplement [all fields] or Dietary Supplementations [all fields] or Food Supplementations [all fields] or Food Supplements [all fields] or Food Supplement [all fields]) |
| 8 | (Diet, Food, and Nutrition [Mesh] or Nutrition Education [all fields]) |
| 9 | (Nutrition Therapy [Mesh] or Medical Nutrition Therapy [all fields]) |
| 10 | (Feeding Behavior [Mesh] Eating Behavior [all fields] or Eating Behaviors [all fields] or Feeding Patterns [all fields] or Feeding Pattern [all fields] or Food Habits [all fields] or Food Habit [all fields] or Eating Habits [all fields] or Eating Habit [all fields] or Dietary Habits [all fields] or Dietary Habit [all fields] or Diet Habits [all fields] or Diet Habit [all fields]) |
| 11 | (Diet [Mesh] or Diets [all fields]) |
| 12 | (Exercise [Mesh] or Exercises or Physical Activity [all fields] or Activities, Physical [all fields] or Activity, Physical [all fields] or Physical Activities [all fields] or Exercise, Physical [all fields] or Exercises, Physical [all fields] or Physical Exercise [all fields] or Physical Exercises [all fields]) |
| 13 | (Multifactorial Intervention [all fields] or Multifactorial Interventions [all fields]) |
| 14 | 7 or 8 or 9 or 10 or 11 or 12 or 13 |
| 15 | 1 and 6 and 14  Filters: Clinical Trial; Publication date from 2001/01/01 to 2018/08/21; Humans |

1. **Embase**

| Search # | Search formula |
| --- | --- |
| 1 | 'Frailty'/exp OR Frailties OR Frailness OR (Frailty Syndrome) |
| 2 | ‘Aged’/exp OR Elderly OR Senior |
| 3 | ‘Geriatrics’/exp OR Geriatric |
| 4 | ‘Very Elderly’/exp OR (Aged, 80 and over) OR centenarian OR centenarians OR nonagenarian OR nonagenarians OR octogenarian OR octogenarians OR (very old) |
| 5 | ‘Frail Elderly’/exp |
| 6 | 2 or 3 or 4 or 5 |
| 7 | ‘Nutrition’/exp OR (diet, food, and nutrition) OR (nutrition council) OR (nutrition phenomena) OR (nutrition processes) OR (nutrition study) |
| 8 | ‘Diet therapy’/exp OR (diet treatment) OR (dietary therapy) OR (dietary treatment) OR (nutrition therapy) |
| 9 | ‘Feeding Behavior’/exp OR (alimentary behavior) OR (alimentary behavior) OR (behavior, alimentary) OR (behavior, eating) OR (behaviour, alimentary) OR (behaviour, eating) OR (eating behavior) OR (eating behavior) OR (feeding behavior) OR (feeding habit) OR (feeding pattern) OR (feeding program) OR (feeding programme) OR (feeding time) OR (food habit) OR (food habits) OR (meal time) OR (nutrition habit) OR (nutrition pattern) OR (nutritional habit) |
| 10 | ‘Diet’/exp OR (diet influence) OR (diet regimen) OR (dietary effect) OR (dietary influence) OR dieting |
| 11 | ‘Nutrition education’/exp OR (education, nutrition) OR (food education) OR (nutritional education) |
| 12 | ‘Dietary supplement’/exp OR (diet additive) OR (diet supplement) OR (dietary supplements) OR (food supplement) OR (supplementary diet) |
| 13 | ‘Exercise’/exp OR (biometric exercise) OR (effort; exercise capacity) OR (exercise performance) OR (exercise training) OR (physical effort) OR (physical exercise) |
| 14 | (Multifactorial Interventions) or (Multifactorial Intervention) |
| 15 | 7 or 8 or 9 or 10 or 11 or 12 or 13 or 14 |
| 16 | 1 and 6 and 15 |
| 17 | #16 AND [embase]/lim NOT ([embase]/lim AND [medline]/lim) |
| 18 | #16 AND [embase]/lim NOT ([embase]/lim AND [medline]/lim) AND ('clinical trial'/de OR 'controlled clinical trial'/de OR 'controlled study'/de OR 'human'/de OR 'major clinical study'/de OR 'randomized controlled trial'/de OR 'randomized controlled trial (topic)'/de) AND (2001:py OR 2002:py OR 2003:py OR 2004:py OR 2005:py OR 2006:py OR 2007:py OR 2008:py OR 2009:py OR 2010:py OR 2011:py OR 2012:py OR 2013:py OR 2014:py OR 2015:py OR 2016:py OR 2017:py OR 2018:py) AND ([aged]/lim OR [middle aged]/lim OR [very elderly]/lim) |

1. **Lilacs**

| Search # | Search formula |
| --- | --- |
| 1 | (Frailty OR Fragilidad OR Fragilidade OR Síndrome da Fragilidade) AND (Aged OR Anciano OR Idoso OR Idosos OR Pessoa Idosa OR Pessoa de Idade OR Pessoas de Idade OR Pessoas Idosas OR População Idosa OR Geriatrics OR Geriatría OR Geriatria OR Frail Elderly OR Anciano Frágil OR Idoso Fragilizado OR Idosos Fragilizados OR Idoso Dependente OR Idosos Dependentes OR Adultos Idosos Fragilizados OR Idoso com Deficiência Funcional OR Idoso Débil OR Idoso Debilitado OR Idosos Debilitados) AND (Feeding OR Alimentación OR Alimentação OR Diet OR Dieta OR Regime Alimentar OR Food and Nutrition Education OR Educación Alimentaria y Nutricional OR Educação Alimentar e Nutricional OR Educação Nutricional OR Educação Alimentar OR Supplementary Feeding OR Alimentación Suplementaria OR Suplementação Alimentar OR Alimentação Complementar OR Alimento Complementar OR Suplementação Nutricional OR Suplementação Dietética OR Suplementos Nutricionais OR Suplementos Dietéticos OR Dietary Supplements OR Nutrition Therapy OR Terapia Nutricional OR Terapia Médica Nutricional OR Exercise OR Ejercicio OR Exercício OR Exercício Aeróbico OR Exercício Físico OR Atividade Física para Idoso OR Atividade Física OR Treinamento Físico OR Multifactorial Interventions OR Intervención Multifactorial OR Intervenções Multifatoriais OR Intervenção Multifatorial) AND (Randomized Controlled Trial OR Ensayo Clínico Controlado Aleatorio OR Ensaio Clínico Controlado Aleatório OR Ensaio Controlado Aleatório OR Ensaio Clínico Controlado Randomizado) |

1. **Web of Science**

| Search ID # | Search formula |
| --- | --- |
| 1 | TS= (Frailty or Frailties or Frailness or “Frailty Syndrome”) |
| 2 | TS= (Aged or Elderly or Senior) |
| 3 | TS= (Geriatrics or Geriatric) |
| 4 | TS= (“Aged, 80 and over” or “Oldest Old” or Nonagenarians or Nonagenarian or Octogenarians or Octogenarian or Centenarians or Centenarian) |
| 5 | TS= (“Frail Elderly” or “Frail Elders” or “Frail Elder” or “Functionally-Impaired” or Elderly or “Functionally Impaired Elderly” or “Frail Older Adults” or “Frail Older Adult”) |
| 6 | 2 or 3 or 4 or 5 |
| 7 | TS= (“Dietary Supplements” or “Dietary Supplement” or “Dietary Supplementations” or “Food Supplementations” or “Food Supplements” or “Food Supplement”) |
| 8 | TS= (“Diet, Food, and Nutrition” or “Nutrition Education”) |
| 9 | TS= (“Nutrition Therapy” or “Medical Nutrition Therapy”) |
| 10 | TS= (“Feeding Behavior” or “Eating Behavior” or “Eating Behaviors” or “Feeding Patterns” or “Feeding Pattern” or “Food Habits” or “Food Habit” or “Eating Habits” or “Eating Habit” or “Dietary Habits” or “Dietary Habit” or “Diet Habits” or “Diet Habit”) |
| 11 | TS= (Diet or Diets) |
| 12 | TS= (Exercise or Exercises or “Physical Activity” or “Activities, Physical” or “Activity, Physical” or “Physical Activities” or “Exercise, Physical” or “Exercises, Physical” or “Physical Exercise” or “Physical Exercises”) |
| 13 | TS= (“Multifactorial Interventions” or “Multifactorial Intervention”) |
| 14 | 7 or 8 or 9 or 10 or 11 or 12 or 13 |
| 15 | TS= (“Randomized Controlled Trial” or “Controlled Clinical Trial” or “Clinical Trial” or “Intervention Study”) |
| 16 | 1 and 6 and 14 and 15  Stipulated time: 2001-2018. |

1. **Cinahl**

| Search ID # | Search formula |
| --- | --- |
| S1 | TX (Frailty or Frailties or Frailness or Frailty Syndrome) |
| S2 | TX (Aged or Elderly or Senior) |
| S3 | TX (Geriatrics or Geriatric) |
| S4 | TX (Aged, 80 and over or Oldest Old or Nonagenarians or Nonagenarian or Octogenarians or Octogenarian or Centenarians or Centenarian) |
| S5 | TX (Frail Elderly or Frail Elders or Frail Elder or Functionally-Impaired or Elderly or Functionally Impaired Elderly or Frail Older Adults or Frail Older Adult) |
| S6 | S2 or S3 or S4 or S5 |
| S7 | TX (Dietary Supplements or Dietary Supplement or Dietary Supplementations or Food Supplementations or Food Supplements or Food Supplement) |
| S8 | TX (Diet, Food, and Nutrition or Nutrition Education) |
| S9 | TX (Nutrition Therapy or Medical Nutrition Therapy) |
| S10 | TX (Feeding Behavior or Eating Behavior or Eating Behaviors or Feeding Patterns or Feeding Pattern or Food Habits or Food Habit or Eating Habits or Eating Habit or Dietary Habits or Dietary Habit or Diet Habits or Diet Habit) |
| S11 | TX (Diet or Diets) |
| S12 | TX (Exercise or Exercises or Physical Activity or Activities, Physical or Activity, Physical or Physical Activities or Exercise, Physical or Exercises, Physical or Physical Exercise or Physical Exercises) |
| S13 | TX (Multifactorial Interventions or Multifactorial Intervention) |
| S14 | S7 or S8 or S9 or S10 or S11 or S12 or S13 |
| S15 | S1 AND S6 AND S14  **Limiters** – Publication date: 20010101-20181231; Delete records from MEDLINE; Human; Publication type: Randomized Controlled Trial |

1. **Cochrane**

| Search ID # | Search formula |
| --- | --- |
| 1 | (Frailty or Frailties or Frailness or “Frailty Syndrome”) |
| 2 | (Aged or Elderly or Senior) |
| 3 | (Geriatrics or Geriatric) |
| 4 | (“Aged, 80 and over” or “Oldest Old” or Nonagenarians or Nonagenarian or Octogenarians or Octogenarian or Centenarians or Centenarian) |
| 5 | (“Frail Elderly” or “Frail Elders” or “Frail Elder” or “Functionally-Impaired” or Elderly or “Functionally Impaired Elderly” or “Frail Older Adults” or “Frail Older Adult”) |
| 6 | 2 or 3 or 4 or 5 |
| 7 | (“Dietary Supplements” or “Dietary Supplement” or “Dietary Supplementations” or “Food Supplementations” or “Food Supplements” or “Food Supplement”) |
| 8 | (“Diet, Food, and Nutrition” or “Nutrition Education”) |
| 9 | (“Nutrition Therapy” or “Medical Nutrition Therapy”) |
| 10 | (“Feeding Behavior” or “Eating Behavior” or “Eating Behaviors” or “Feeding Patterns” or “Feeding Pattern” or “Food Habits” or “Food Habit” or “Eating Habits” or “Eating Habit” or “Dietary Habits” or “Dietary Habit” or “Diet Habits” or “Diet Habit”) |
| 11 | (Diet or Diets) |
| 12 | (Exercise or Exercises or “Physical Activity” or “Activities, Physical” or “Activity, Physical” or “Physical Activities” or “Exercise, Physical” or “Exercises, Physical” or “Physical Exercise” or “Physical Exercises”) |
| 13 | (“Multifactorial Interventions” or “Multifactorial Intervention”) |
| 14 | 7 or 8 or 9 or 10 or 11 or 12 or 13 |
| 15 | 1 and 6 and 14  In trials  Year first published: 2001 to 2018 |
